# The curcumin analog (PAC) suppressed cell survival and induced apoptosis and autophagy in oral cancer cells

**DOI:** 10.1038/s41598-021-90754-x

**Published:** 2021-06-03

**Authors:** Abdelhabib Semlali, Camille Contant, Basem Al-Otaibi, Ibrahim Al-Jammaz, Fatiha Chandad

**Affiliations:** 1 Groupe de Recherche en Écologie Buccale, Faculté de Médecine Dentaire, Université Laval, Québec, QC G1V 0A6 Canada; 2 Department of Cyclotron and Radiopharmaceuticals, King Faisal Specialist Hospital and Research Center, MBC≠03, P.O. Box 3354, Riyadh, 11211 Saudi Arabia

**Keywords:** Targeted therapies, Cancer therapy, Head and neck cancer, Cancer, Cell biology, Molecular biology, Plant sciences

## Abstract

PAC (3,5-*Bis* (4-hydroxy-3-methoxybenzylidene)-*N*-methyl-4-piperidone), a novel bioactive curcumin analog, has been reported to have anticancer properties against various tumors. However, the anti-cancer effects of PAC on oral cavity squamous cell carcinoma were not studied yet. Our aim is to investigate the anti-oral cancer properties of PAC in vitro, and determine the molecular mechanisms underlying these effects. Viability assays including MTT and LDH were conducted to measure cell proliferation. Flow cytometry-based cytotoxicity assay was performed to detect autophagic cell death and oxidative stress markers. Western blotting was used for measuring protein expression/activation in apoptotic, autophagic and pro-carcinogenic cellular signaling pathways. We demonstrated that PAC preferentially and, in a dose, -dependent way kills oral cancer cells, but was not toxic to normal human gingival cells. PAC destabilizes cell-cycle distributions, inhibits the expression of oncogenes (cyclin D1) and that of cyclin-dependent kinase inhibitor (p21^WAF1^) is upregulated, increases the expression of *p53* gene, and inhibits epithelial-mesenchymal transition markers in oral cancer cells. The PAC effect involve various signaling pathways including NF-κB, MAPK, Wnt, caspase-3/9 and PARP1. Finally, PAC demonstrated ability to induce autophagy, decrease production of reactive oxygen species, increase intracellular glutathione (GSH) activity, and reduce mitochondrial membrane potential in oral cancer cells. In conclusion, PAC inhibits the proliferation and increases the apoptosis and autophagy and oxidative stress of oral cancer cells. These effects involve ERK1/2, p38/JNK, NF-κB and Wnt cellular signaling pathways. Overall, our study suggests the potential use of PAC to treat oral cancer.

## Introduction

Oral cancer is commonly known as one of the head and neck cancers. It is the sixth most common malignancy worldwide^1^. According to the Canadian Cancer Society in 2019, approximately 5300 people will be diagnosed with oral cavity cancer, which 3700 cases concerned men and 1600 are women. This cancer will cause death of 1450 Canadians, which 1050 are men and 450 women^2^. Oral cancer is critical, because the patient may not notice it in its first stages, as it can frequently thrive without generating pain or symptoms, and generally, it produces primary tumors. This means that patients, who survive a first encounter with the disease, have up to 20 times higher risk of developing a second cancer^3^. Largely, those who have been suffering from buccal mucosa cancer for 5 years have about a 50 percent life expectancy or mortality rate^4^).

Treating oral cancer conventionally is highly dependent on surgery assisted with or without chemotherapy and radiotherapy. For destroying malignant cells, chemicals are used more extensively as a systemic treatment^5^. The panel of anti- cancer drugs includes mainly 5-fluorouracil, hydroxyurea, platinum derivatives, anthracyclines, plant alkaloids^6^. The most common chemotherapy medicines consists of cisplatin^7^. Despite its survival benefits as an anticancer drug, it has side effects, especially the chemoresistance that is limiting patients in clinical effectiveness^7^. This knowledge has inspired the use of herbal medicinal products as complementary and/or alternative therapies to a direct remedy, thus the recognition of plant products as effective and inexpensive sources of novel synthetic chemotherapeutic compounds is increasing. For decades, in some countries such as China, India, and Egypt, traditional medicine has been making use of flavored herbs in its home-based therapies for many diseases, including cancer. Nowadays, it is becoming more widely acceptable to consider herbal remedies as helpful supplements to medical prescriptions^8,9,10^.

Curcumin, a yellow shade from *Curcuma longa* Linn., is a significant part of turmeric and is normally used as a flavor and nourishment shading material^11^). Furthermore, various in vitro and in vivo (animal) studies suggest that Curcumin and derivatives have extensive biological activity as an antioxidant, neuroprotective, antitumor, anti-inflammatory^12–15^. Curcumin can rummage around scavenging various types of free radicals, for example, reactive oxygen and nitrogen species (ROS and RNS, individually), it can adjust movement of GSH, catalase, and SOD enzymes active in their neutralization^16^. Additionally, curcumin can inhibit ROS-producing enzymes lipoxygenase / cyclooxygenase and xanthine dehydrogenase / oxidase^16^. Curcumin has been studied in numerous human carcinomas^17^. The mechanisms by which it exerts its anticancer effects are diverse, targeting many levels of regulation involved in processes of cellular growth and apoptosis^18^. Other than the curcumin’s effects on different transcription factors, oncogenes and signaling proteins^17,18,19^ it also acts at various temporal stages of carcinogenesis^17^. Some of its molecular targets that have been explored for cancer treatment are the effect of curcumin on cyclin-CDK complexes and CDK inhibitors as well as p53 pathway^20^ and MAP kinase, Wnt/β-catenin signaling pathways^20^. Despite the fact that curcumin has been successfully evaluated for wide-ranging biological activities, the two main concerns related to its poor bioavailability and fast metabolization incited scientists to search for novel synthetic analogs in order to overcome these drawbacks and to gain in efficacy. Iten M. Fawzy and his colleagues (2014) synthesized different curcumin analogs including PAC, 3,5-*Bis* (4-hydroxy-3-methoxybenzylidene)-*N*-methyl-4-piperidone^21,22^) Another derivative 5-*Bis* (4-hydroxy-3-methoxybenzylidene)-*N*-methyl-4-piperidone, represses expansion through delaying the cell cycle at G2/M phase both in breast and colorectal cancer cells. Furthermore, it was established that PAC is also repressing NF-kB and its downstream effectors cyclin D1 and Bcl-2 (B-cell lymphoma 2) as well as JAK2/STAT3, AKT/mTOR (mammalian target of rapamycin) and MEK/ERK (mitogen-activated protein kinase/extracellular signal-regulated kinase) signaling pathways^21,23,24^. It was demonstrated that PAC is a potent inhibitor of these pathways and has better solubility and biodistribution than curcumin^25^. We suggested that, PAC causes preferential destructive effects on oral cancer cells by way of oxidative stress and DNA damage. Therefore, PAC does have chemopreventative and chemotherapy potential, for having antitumor and antioxidant properties that subvert chemoresistance and reduce nonspecific toxicity in normal cells, as do Cisplatin -based therapies used in the fight against oral cancer. Our general aim is to investigate in vitro, the role of PAC in inhibiting of oral cancer cell proliferation. Our two principal objectives were the following: (1) to investigate the mechanisms behind the ant proliferative effects of PAC on Ca9-22; and, (2) to study the effects of PAC on cell oxidative stress and DNA damage. Until today, no study was available to investigate its properties to possibly treat oral cancer. This research is aiming at studying the inhibitory effects of the curcumin analog PAC on oral cell proliferation and apoptosis, as well as investigating the signaling pathways involved in the interactions of PAC with oral cancer cells.

## Materials and methods

### Reagents

5-*Bis* (4-hydroxy-3-methoxybenzylidene)-*N*-methyl-4-piperidone (PAC) was obtained from Dr. Ibrahim Al-Jammaz’s laboratory (Riyadh, Saudi Arabia). Ca9-22 cell line was purchased from RIKEN BioResource Research Center (Tsukuba-shi, Japan). However, primary human gingival epithelial cells (GEC) and primary gingival fibroblasts (GF), were isolated from healthy human gingival tissues as we previously reported. The Ethical committee of University Laval approved the use of primary human gingival cells. The gingival cells were extracted after the donor informed written consent**.** All methods were performed in accordance with the relevant guidelines and regulations by including a statement.

RPMI-1640 (Corning, cat ≠ 10-040-CV, F12, DMEH cell culture media (Corning, cat ≠ 10-013-CV), fetal bovine serum (FBS) (Thermo Fisher, cat ≠ 10,099-141), Hoechst 33,342 (ThermoFisher, cat ≠ 62,249 and MitoSOX Red Mitochondrial Superoxide Indicator have been bought from Thermo Fisher Scientific (Thermo Fisher Scientific, Burlington, ON, Canada, cat ≠ M36008). Penicillin–Streptomycin (Gibco, cat ≠ 15,140-122), MTT (Sigma, cat ≠ C501/CT02) and LDH colorimetric assays (Roche, cat ≠ 11,644,793,001), Trypsin–EDTA solution (Sigma, cat ≠ 59417C), Caspases detection kit (FITC-VAD-FMK) (Sigma,cat ≠ QIA90-1Kit) and β-actin antibody (Sigma, Oakville, Ontario, Canada, cat ≠ A5441)). FITC Annexin V Detection Kit I with propidium iodide was obtained from Biolegends (Saint Diego, CA, USA,c at ≠ 640,932). Autophagy Assay (Red) (cat ≠ 9157, Intracellular Total Reactive Oxygen Species (ROS) A ctivity Assay (cat ≠ 9144) and Intracellular Glutathione (GSH) Assay (cat ≠ 9137) were purchased from ImmunoChemistry Technologies (Burlington, ON, Canada). Coomassie Brillant Blue G-250 Dye has been bought from Bio-Rad (Mississauga, ON, Canada, cat ≠ 1,610,406), while PVDF membranes were from GE Healthcare Life Sciences (Oakville, Ontario, Canada, cat 10,600,023). The following antibodies, among which p53 (sc-263), cyclin D1 (sc-8396), p21 (sc-6246), Bcl-2 (sc-509), Bax (sc-7480), PARP-1 (sc-8007), caspase-3 (sc-56046), caspase-9 (sc-17784), NF-κB (sc-8008) and β-catenin (sc-59737) were all obtained from Santa Cruz Biotechnology (Santa Cruz, CA, USA). Anti-vimentin antibody (Ab8978) from Abcam (Cambridge, MA, USA) as well as others to cleaved caspase-3 (9664S), cleaved caspase-9 (20750S), cleaved PARP-1 (5625S), cytochrome C (11940S), E-cadherin (8834), pERK1/2 (4370), ERK1/2 (4695), pp38 MAPK (4631), p38 MAPK (9212), p-SAPK/JNK (4668P), SAPK/JNK (9252S), LC3B (2775) and SQSTM1/p62 (39,749) were all from Cell Signaling Technology (Danvers, MA, USA). The goat anti-mouse (554,002) and anti-rabbit (554,021) secondary and have been bought from BD Pharmingen (Mississauga, ON, Canada).

#### Cell culture and PAC stimulation

Ca9-22 cells and GEC were sub-cultured respectively in RPMI-1640, F12 or DMEM media supplemented with 5% (v/v) FBS for Ca9-22 and 10% for GEC, 100 units/mL penicillin, and 100 µg/mL streptomycin in T75 flasks^26,27^. All cells were maintained in a humidified atmosphere of 5% CO_2_ at 37 °C. All experiments conducted with GEC were at passages 3 to 4, and GF at passages 4 to 5. PAC was used at (0, 1, 2.5, 5 and 10 μM in DMSO) with all experiments, except for Western blotting where we used PAC at 5 μM, only.

#### Cell viability assay

Cells were seeded at 3 × 10^5^ cells/well in 6-well cell culture plates, and culture for 24 h. Culture were then stimulated exposed to vehicle control (DMSO) or various concentrations of PAC (0, 1, 2.5, 5 and 10 μM) for 24 h. At the end of the stimulation period, the culture medium of each condition was supplemented with MTT solution at 0.5 mg/mL then incubated for 3 h at 37 °C in the dark. The cells were washed, then 1 ml of 0.04 N HCl in isopropanol was added to the culture wells, followed by an extended 15-min incubation. Finally, 200 µl of the reaction mixture (in triplicate) was transferred to the wells of a 96-well flat-bottom plate and the absorbance was measured at 550 nm using iMark reader (Bio-Rad). The cell viability (percentage) was determined by using the following formula: % of cell viability = [(OD_550 nm_ (treated cell)/(OD (control cell)] × 100. This experiment was repeated 7 times. To support the cell viability assay we performed lactate dehydrogenase (LDH) activity measurement using culture supernatants. Supernatants were collected from each condition after exposure or not of the cells to PAC for 24 h. The LDH activity was measured by means of an LDH kit from (Sigma-Aldrich), as outlined in our previous work^28^. The experiment was repeated 4 independent times.

#### Colony formation assay

Ca9-22 cells were seeded into 6-well plates at 10^3^ cells per well for 24 h, before being treated with various concentrations of PAC. The culture medium was changed every 2 or 3 days. After 2 weeks, the cell clones that survived were fixed by 100% ethanol for 10 min. After washing twice by PBS, cells were then stained with 0.5% crystal violet for 30 min and they were photographed with a digital camera.

#### Annexin V-FITC apoptosis assay with propidium iodide

Ca9-22 cells were seeded into T25 flasks the exposed to 1 µM, 2.5 µM, 5 µM or 10 µM of PAC, for 24 h and 48 h, at 37 °C. After exposure, cells were detached from the flasks using 0.05% trypsin and 0.01% EDTA, centrifuged, washed by PBS and the pellet was suspended in 300 µl phosphate-buffered saline (PBS) and incubated with 0.5 µl annexin V-FITC and 0.5 µl of propidium iodide at room temperature for 30 min in the dark. Stained cells were subjected to flow cytometry analyses, using a “LSRII” or “CantoII” cytometer instrument from BD Biosciences. Data analysis was made by FACS Diva software v. 6.1.3. The experiments were repeated 5 independent times.

#### Cell cycle distribution

Cell percentage depending on the various phases of the cell cycle was measured by using the fluorescent DNA dye 7-aminoactinomycin D (7-AAD) (Sigma, USA), as previously described^29^. Briefly, oral cancer cells were treated with different PAC concentrations (1 µM, 2.5 µM, 5 µM or 10 µM) for 24 h. They were then incubated in the presence of 7-AAD at 1 μg/mL for 30 min at 37 °C. The fluorescence intensities of DNA were detected with “LSRII” or “CantoII” (BD Biosciences). Data were analyzed by FACSDiva software v. 6.1.3 and experiments were done in triplicates, n = 5.

#### Cell autophagy

To determine the in vitro effect of PAC on Ca9-22 cells autophagy in, we used ICT’s Autophagy Assay (Red) from ImmunoChemistry Technologies. Briefly, Ca9-22 cells were stimulated or not with various concentrations of PAC for 24 h, detached from the culture plates, then incubated with a diluted Autophagy Probe, Red at 1:50 for 1 h in the dark, washed three times with the cellular assay buffer. Stained cells were afterwards analyzed by flow cytometry, (n = 4).

#### Cell oxidative stress

Expression of cellular ROS was evaluated by using a specific assay kit from ImmunoChemistry Technologies. In short, Ca9-22 cells were seeded and cultured from 24 h, then were exposed to different concentrations (1 µM, 2.5 µM, 5 µM or 10 µM) of PAC for 24 h. Total ROS Green dye molecule was added to 10^6 ^cells/mL of each culture condition for 1 h in the dark at 37 °C before making the analysis with BD Accuri C6 Plus Flow Cytometry System (BD Biosciences). For evaluating levels of intracellular GSH, Thio Bright Green reagent was first reconstituted in DMSO, added to the cell suspension (1:200), and then incubated for 30 min in the dark. After washing with PBS twice, fluorescence intensity was determined by Flow Cytometry System (BD Biosciences), and the percentage of GSH-positive cells was calculated. Experiments were done in triplicates.

#### Mitochondrial membrane potential (MMP)

MMP expression reduced by PAC was measured by DiOC_(2)_(3)-based flow cytometry. Ca9-22 cells (10^6^) were seeded into a T325 flask. After overnight incubation for adherence, cells were treated with different concentrations of PAC (0, 1, 2.5, 5 and 10 µM). After 24 h of treatment, 50 nM of DiOC_2_(3) were added to each condition for 30 min. Cells were collected and subjected to flow cytometry analyses using a “LSRII” or “CantoII” cytometer instruments from BD Biosciences. Data analysis was made by FACSDiva software v. 6.1.3., (n = 4).

#### Assessment of mitochondrial superoxide

To evaluate the mitochondrial superoxide production following treatments with PAC, we used an indicator named MitoSOX Red (Molecular Probes, Invitrogen), according to the manufacturer’s instructions, and afterwards performed a flow cytometry analysis. PAC-treated cells (1µ M, 2.5 µM, 5 µM or 10 µM) were incubated for 30 min with 5 μM MitoSOX at 37 °C in 5% CO_2_. They were then suspended in 1 mL PBS, and analyses by means of flow cytometry with “LSRII” or “CantoII” (BD Biosciences) (n = 3).

#### Western blotting

The effect of PAC at 5 µM on various proteins involved in MAPK pathways, apoptosis, autophagy, and cell cycle was evaluated by making use of Western blots. Briefly, 10^6^ cells (stimulated and unstimulated) were harvested for extraction by RIPA lysis buffer. Protein concentrations were determined by the Bradford assay. For each sample, a mixture of 20 μg to 60 μg was added to an equal amount of loading buffer containing 10% β-mercaptoethanol and migrated through a 7–15% acrylamide gel. After electrophoretic separation, proteins were transferred from the gel to a nitrocellulose membrane to be blocked in a 5% milk solution for 1 h at 24 °C and put overnight in an incubator with appropriate concentrations of antibodies. Finally, a band detection was performed by an ECL Imaging System (EMD, Millipore, Billerica, MA, USA), according to instructions from its manufacturer.

#### Statistical analysis

All our experiments were repeated more than three times and the statistical significance obtained between controls (untreated cells) and tests (treated) with various concentrations of PAC was expressed as mean ± SD. Differences between values were determined if statistically significant using a one-way ANOVA. A *p*-value < 0.05 was defined as significant.

## Results

### PAC selectively inhibits the growth of oral cancer cells, and increases apoptosis

**Figure 1:**
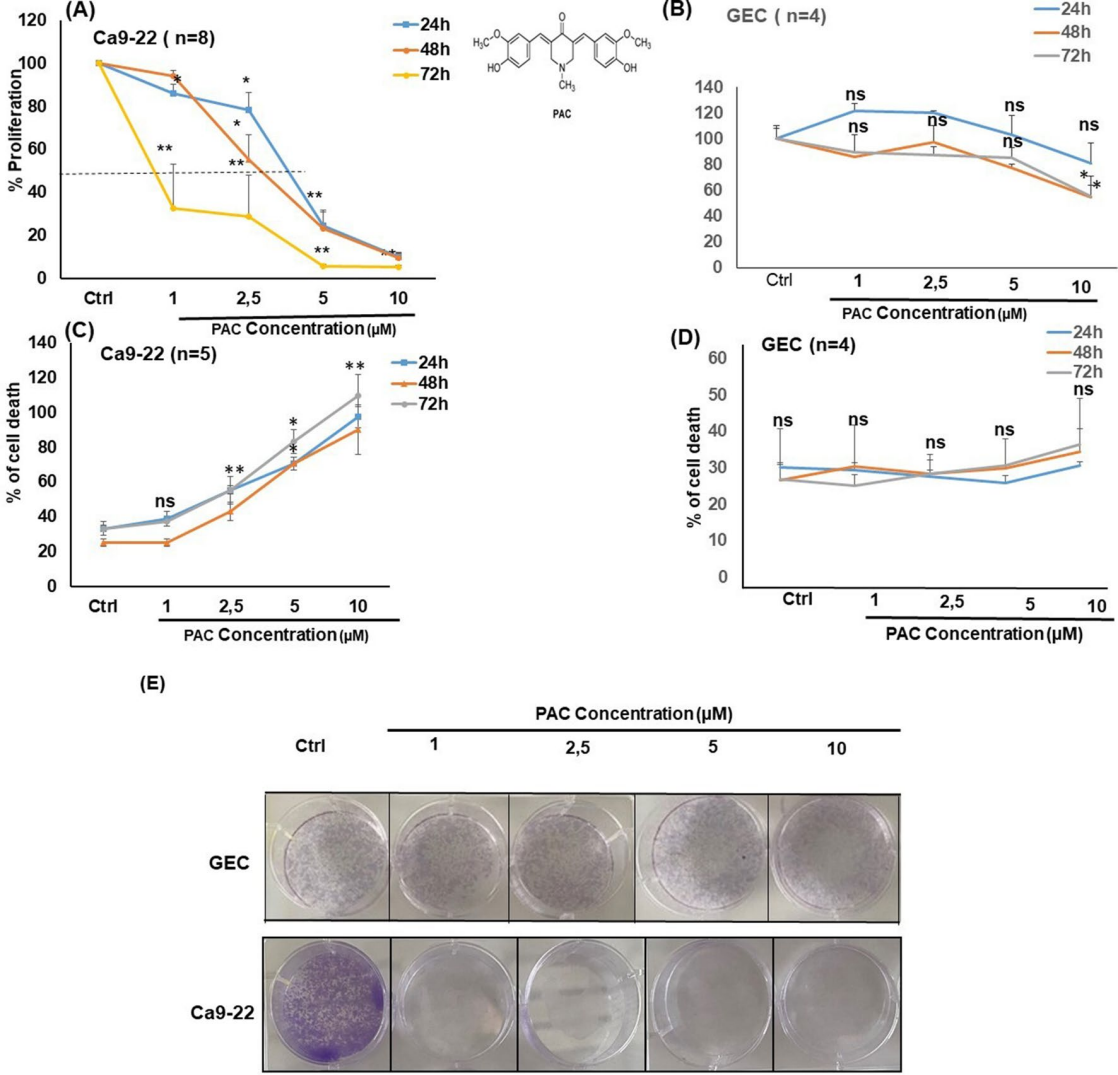
PAC have preferentially ant proliferative activity and cytotoxicity against Ca9-22 cells, but not on normal human gingival epithelial cells. (**A**) PAC at various concentrations decrease the growth of oral cancer cells. This decrease was more important with higher concentration of PAC (n = 7). (**B**) The normal human gingival epithelial cells (GEC) cells, a slight decrease was observed only at the highest concentration of PAC (10 μM) after 48 h (*p* = 0.02) and 72 h (*p* = 0.03) (n = 4). (**C**) A treatment with PAC was inducing dose- and time-dependent cell death in Ca9-22. (**D**) Lactate dehydrogenase (LDH) assay: PAC don’t induce cell death in GEC cells (n = 4). (**E**) PAC inhibits ca9-22 colony formation but not in GEC. The cells were treated with various concentrations of PAC. The culture medium was changed every 2 or 3 days. After 2 weeks, the cell clones that survived were fixed by 100% ethanol for 10 min. After washing twice by PBS, cells were then stained with 0.5% crystal violet for 30 min and they were photographed with a digital camera (n = 4).

**Figure 2:**
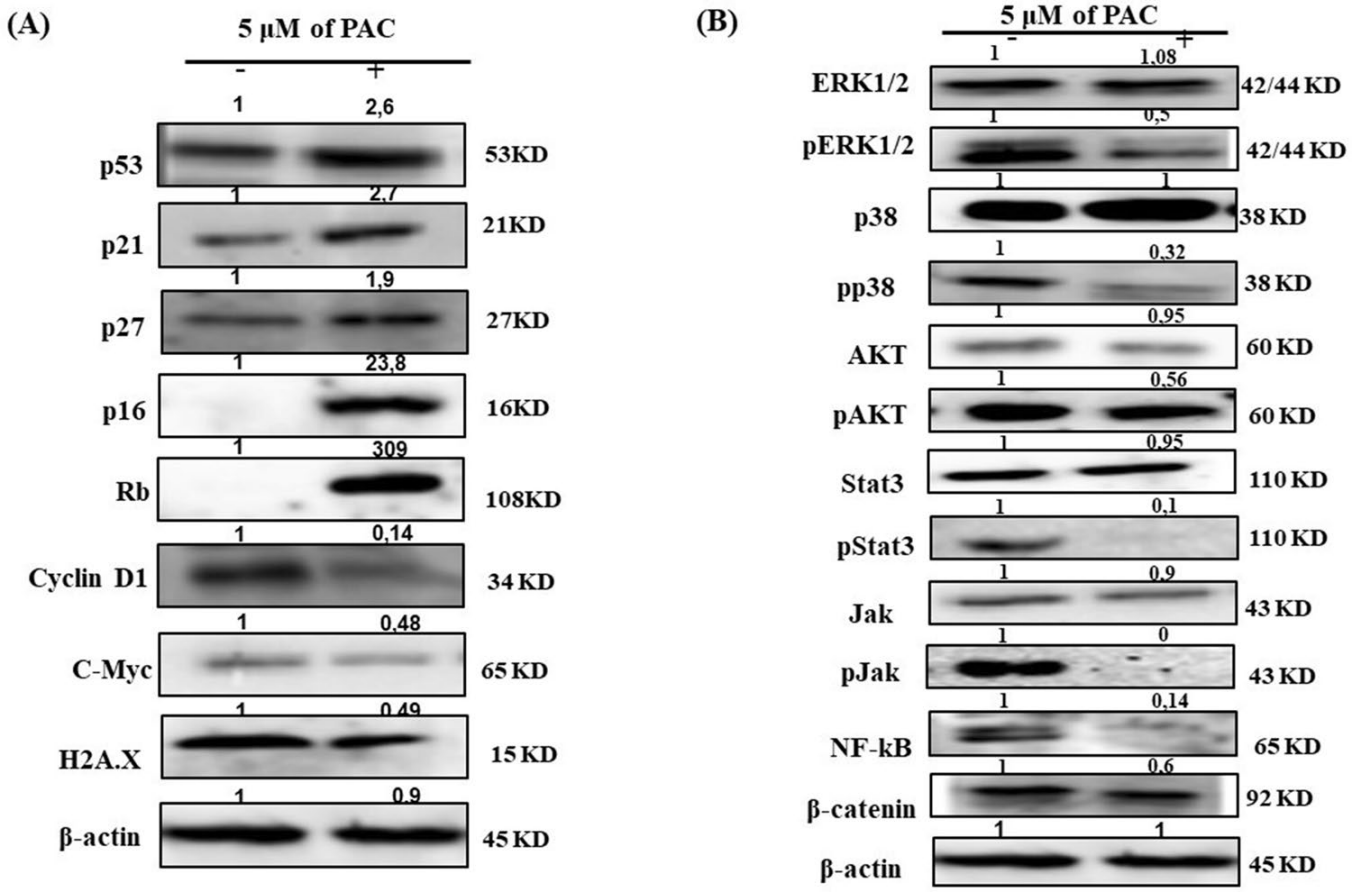
PAC exhibits potent anti-oral cancer properties through targeting several cancer-promoting pathways. (**A**) 5 µM of PAC treatment induces cell-cycle arrest, by increasing the expression of cyclin-dependent kinase inhibitors (CDKIs) such as p21, p27, p16 or p53 and Rb, while it inhibits oncogenes such as cyclin D1 or c-Myc on ca9-22 cells. Also, PAC can to reduce DNA damage on oral cancer cells by inhibition of H2A.X protein expression. (**B**) The effect of PAC on cancer cells is involving specific inhibition of cell proliferation signaling pathways (ERK1/2 phosphorylation, p38, STAT and AKT phosphorylation, NF-κB and Wnt/β-catenin) (n = 3). All Blots for each protein derive from the same experiment and were exposed at same time of phosphor imager exposition.

To evaluate the effect of PAC on normal and gingival cells affected by cancer to proliferate, we used the MTT assay. As presented in Fig. 1, PAC at various concentrations decrease the growth of oral cancer cells. This decrease was more important with higher concentration of PAC. Indeed, at 10 µM oral cancer cell growth was almost drastically reduced. Interesting, the decrease of normal human gingival epithelial cells growth following the exposure to 10 uM of PAC was too low compared to cancer cells. With 5 µM of PAC, there was an 80% growth reduction of oral cancer cells, but only 10% to 20% growth reduction of primary human gingival epithelial cells. These results demonstrated that normal human cells exhibit great resistance to PAC, in comparison to Ca9-22 cells. The effect of PAC on cancer cells was observable even at low concentration with an LC_50_ is around 3 µM. Its proliferation level decreased, from 86.21% ± 4.03% (*p* = 0.007), 78.41% ± 8.23% (*p* = 0.02), 24.37% ± 7.31% (*p* = 2.39 × 10^−5^) to 9.85% ± 2.16% (*p* = 6.46 × 10^−9^) respectively with PAC concentrations of 1, 2.5, 5 or 10 μM (Fig. 1A). With GEC cells, a slight decrease was observed only at the highest concentration of PAC (10 μM) after 48 h (*p* = 0.02) and 72 h (*p* = 0.03), but any significant inhibition for all treatments (1 μM to 10 μM) was noticeable after 24 h. (Fig. 1B). These outcomes were confirmed using a cytotoxicity assay (Fig. 1C,D), a treatment with PAC was inducing dose- and time-dependent cell death in Ca9-22 (Fig. 1C) but not in GEC cells (Fig. 1D), suggesting a selective toxic effect of PAC on cancer rather than normal gingival epithelial cells. We also demonstrated a significant effect of PAC reducing the capacity of oral cancer cells to form colony but not in GEC (Fig. 1E). These outcomes clearly indicate as well that PAC is having preferentially anti-proliferative activity and cytotoxicity against Ca9-22 cells, but not on normal human gingival epithelial cells. We demonstrated that anti-proliferative effect of PAC on oral cancer cells goes through a cell cycle modification. Indeed, Ca9-22 cells treated with 5 µM showed a cell-cycle arrest, by increasing the expression of cyclin-dependent kinase inhibitors (CDKIs) such as p21, p27, p16 or p53 and Rb (retinoblastoma protein) which are tumor suppressor genes known to be inactive in most cancer types, while it inhibits oncogenes such as cyclin D1 or c-Myc. In addition, PAC can to reduce DNA damage on oral cancer cells by inhibition of H2A.X protein expression (Fig. 2A). The effect of PAC on cancer cells is involving specific inhibition of cell proliferation signaling pathways. Indeed, we demonstrated a significant decrease of ERK1/2 phosphorylation, p38, STAT and AKT phosphorylation. Also, PAC at 5 µM was decreasing the expression of NF-kB and Wnt/β-catenin signaling pathways (Fig. 2B).

### Effect of treatments with PAC on cell cycle distribution in oral cells

**Figure 3:**
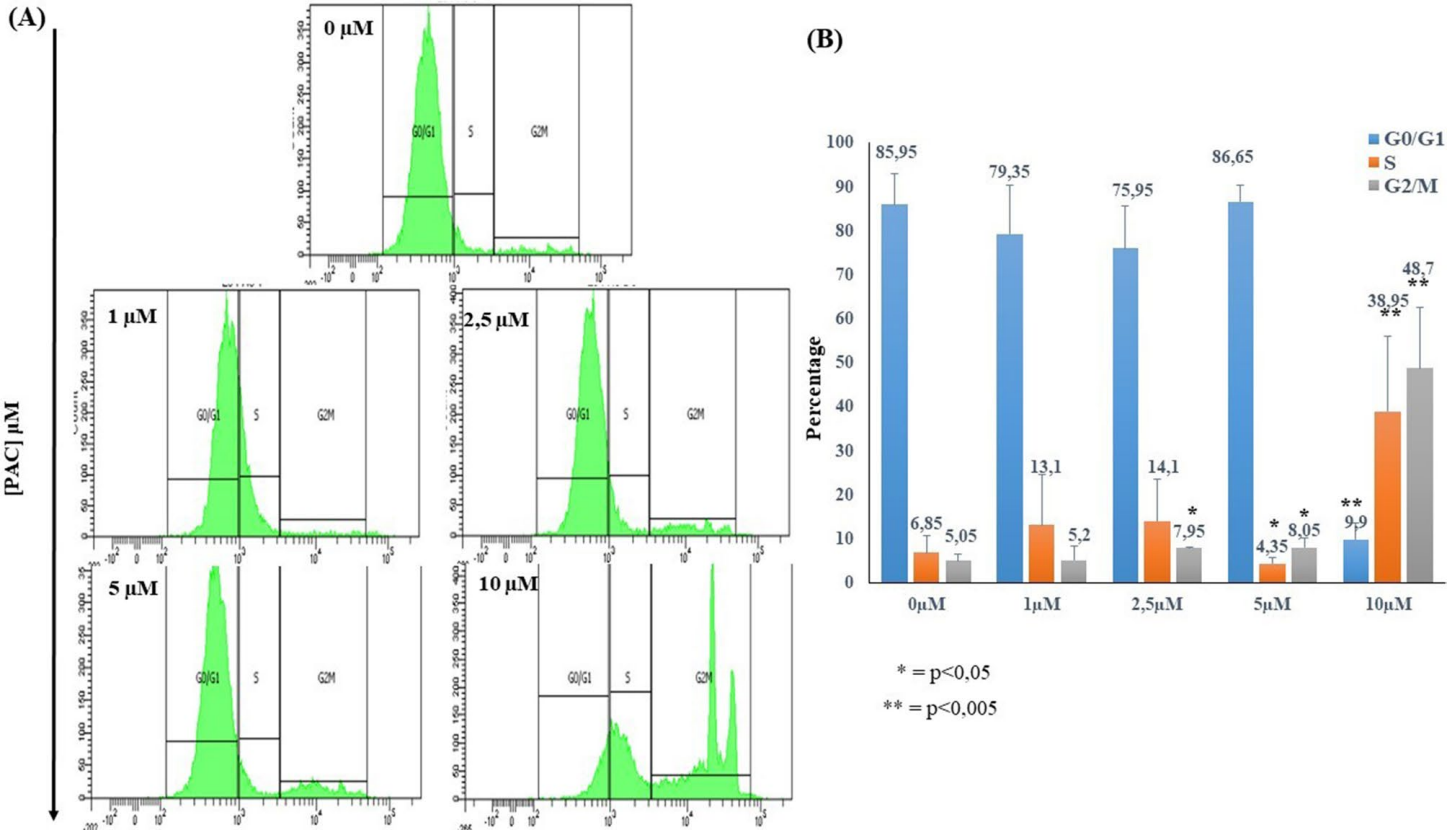
Effect of treatments with PAC on cell cycle distribution in oral cells. (**A**) Cell cycle distribution was evaluated by flow cytometry using the fluorescent DNA dye 7-aminoactinomycin D (7-AAD) (n = 5). (**B**) Statistics analysis of cell cycle distribution in Fig. 3A. Ca9-22 were treated with different PAC concentrations for 24 h. Then, they were responding to PBS buffer before being incubated with 7-AAD**.** Our results shown that different concentrations of PAC were altering Ca9-22 cell cycle distribution.

Cell cycle distribution, on Ca9-22 PAC-treated cells, was evaluated by flow cytometry using the fluorescent DNA dye 7-aminoactinomycin D (7-AAD). Briefly, these were under treatment with different PAC concentrations for 24 h. Then, they were responding to PBS buffer before being incubated with 7-AAD Fig. 3 is presenting that different concentrations of PAC were altering Ca9-22 cell cycle distribution. However, percentage of G0/G1 population decreases dramatically, from 85.95% ± 7.14% in untreated cells to 9.9% ± 3.67%, *p* < 0.005) in cells treated with 10 µM of PAC. In contrary, the S population increases from 6.85% ± 3.74% in controls to 38.95% ± 17.18%, *p* < 0.005 using the same concentration it. The G2/M population is also doing it as the previous one, from 5.05% ± 1.34% to 48.7% ± 13.85% *p* < 0.005 with the same treatment (Fig. 3A,B).

### PAC induces apoptosis in oral cancer cells by targeting the intrinsic mitochondrial pathway

**Figure 4:**
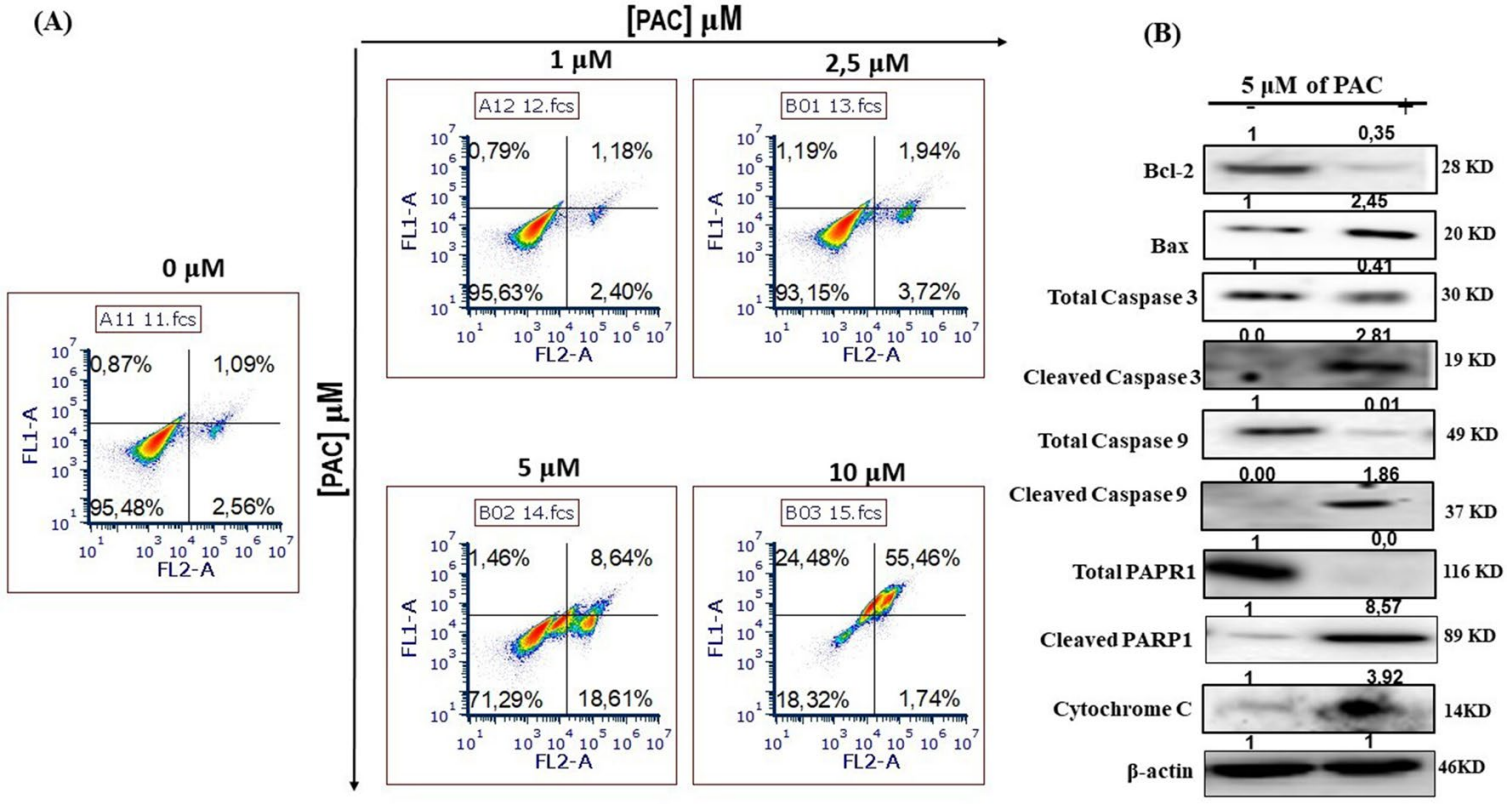
PAC induces apoptosis in oral cancer cells by targeting the intrinsic mitochondrial pathway. (**A**) Fluorochrome-labeled annexin V with PI assay; stained cells were subjected to flow cytometry analyses, using a “LSRII” or “CantoII ” cytometer instrument from BD Biosciences (n = 5). (**B**) PAC dose—dependant increased apoptosis by activation of caspases in Ca9-22 cells (n = 5). (**C**) The effect of PAC of caspase signaling for oral cancer cell apoptosis was confirmed by Western blotting. PAC at 5 µM strongly decreases Bcl-2 expression and increases that of pro-apoptotic Bax and cytochrome C as well as allows cleavage of PARP-1. The effect of PAC promoting cancer cell death involves caspase-3/9 (n = 3). All Blots for each apoptosis protein derive from the same experiment and were exposed at same time of phosphor imager exposition.

In determining the type of death induced by various concentrations of PAC on oral cancer cells, the fluorochrome-labeled annexin V with PI was used. Our results show the presence of 4 different sub-populations referring to live cells (annexin V-/PI-), early apoptotic cells (annexin V + /PI−), late apoptotic cells (annexin V + /PI +) and necrotic cells (annexin V−/PI +). After 24 h of exposure to PAC, the percentage for the first population decreases from 96.48% for untreated to 71.29% with 5 µM, and 18.32% with 10 µM. Conversely, the early apoptotic cells were increasing from 2.56% with the control to 18.62 with 5 µM. In late apoptotic population was much higher with PAC exposure ranging from, 1.09% with the controls to 55.46% with 10 µM of PAC. The necrotic population was also increasing from 0.87% with the untreated cells to 24.48% with 10 µM PAC treated oral cancer cells (Fig. 4A). To confirm apoptosis—inducing ability of PAC concentration on oral cancer cells, we evaluated caspase activation by using flow cytometry. Our results show that PAC dose—dependant increased apoptosis in terms of percentage of positive pancaspase in Ca9-22 cells (Fig. 4B).

The effect of PAC of caspase signaling for oral cancer cell apoptosis was confirmed by Western blotting (Fig. 4C) showing that PAC at 5 µM strongly decreases Bcl-2 expression and increases that of pro-apoptotic Bax and cytochrome C as well as allows cleavage of PARP-1. The effect of PAC promoting cancer cell death involves caspase 3/9 (Fig. 4C).

### PAC promotes autophagy in oral cancer cells by targeting LC3B and p62 protein expression

**Figure 5:**
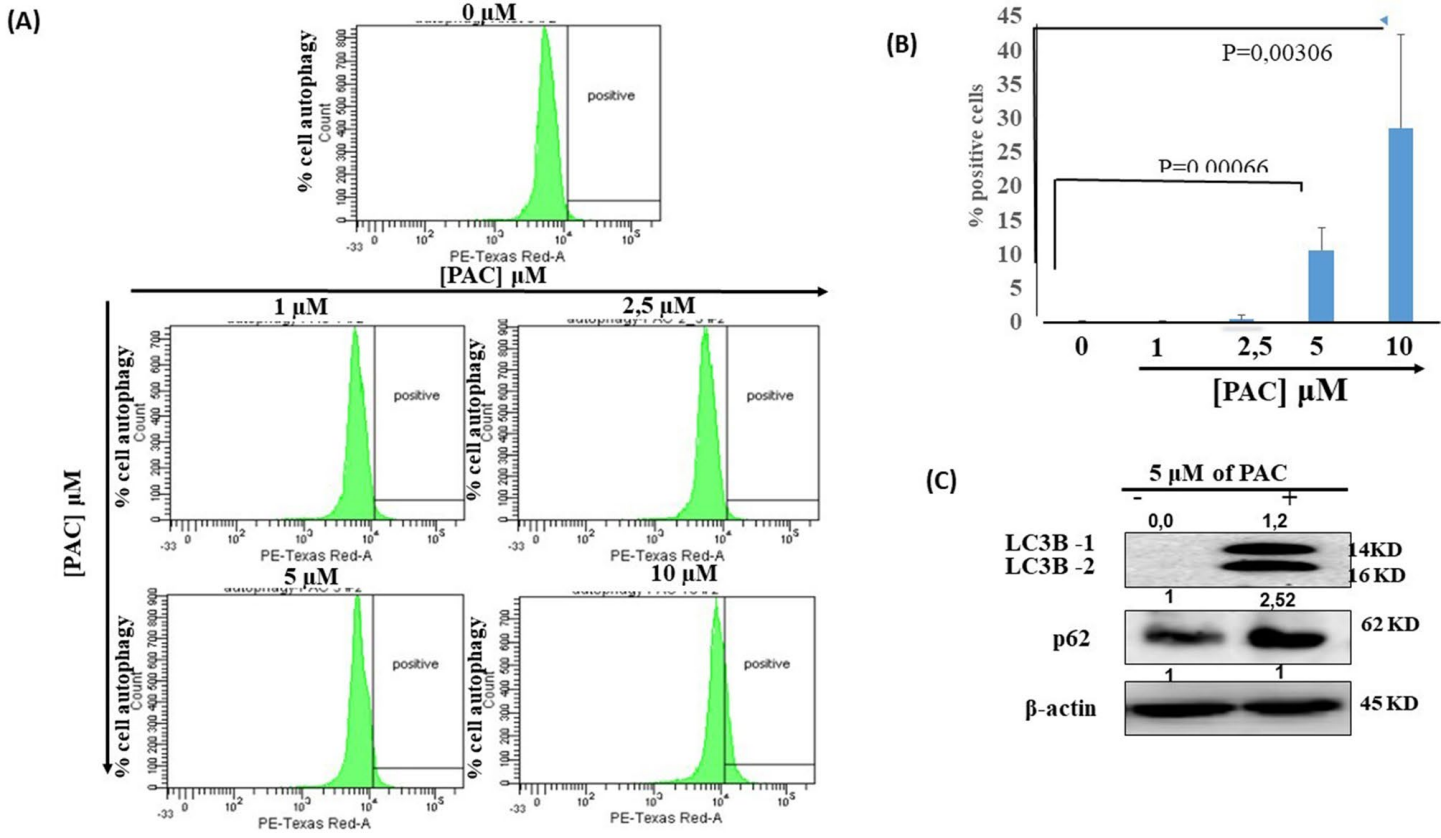
PAC promotes autophagy in oral cancer cells by targeting LC3B and p62 protein expression. (**A**) in vitro effect of PAC on Ca9-22 cells autophagy: Ca9-22 cells were stimulated or not with various concentrations of PAC for 24 h, then incubated with a diluted Autophagy Probe, Red at 1:50 for 1 h in the dark. Stained cells were afterwards analyzed by flow cytometry, (n = 3). (**B**) Statistics analysis of fold changes in % of staining autophagy cells between ca9-22 treated by PAC concentration and Ca9-22 untreated cells (t = test, *p* < 0.005; n = 3). **(C)** Western Blot showing an overexpression at protein levels of two autophagy markers (LC3B and p62) in Ca9-22 cells treated by 5 µM of PAC (n = 3). All Blots for each autophagy protein derive from the same experiment and were exposed at same time of phosphor imager exposition.

To determine the different cellular mechanisms by which PAC exerts its anti-oral cancer function, we investigated the effect of PAC on cell autophagy. As shown in Fig. 5A,B, PAC at 5 μM strongly leads to autophagy in cancer cells. The percentage of positive cells detected by the Autophagy Assay (Red) was increasing from 0.1125% ± 0.15% in controls to 10.57% ± 3.35% (*p* = 0.00066) and 28.59 ± 13.78% (*p* = 0.0030) respectively for 5 µM or 10 µM of PAC. These observations were confirmed by Western Blot showing an overexpression (high protein levels) of two autophagy markers (LC3B and p62) in Ca9-22 cells (Fig. 5C).

### PAC suppresses the process of epithelial-to-mesenchymal transition in oral cancer cells and mediates the anti-metastatic activity

**Figure 6:**
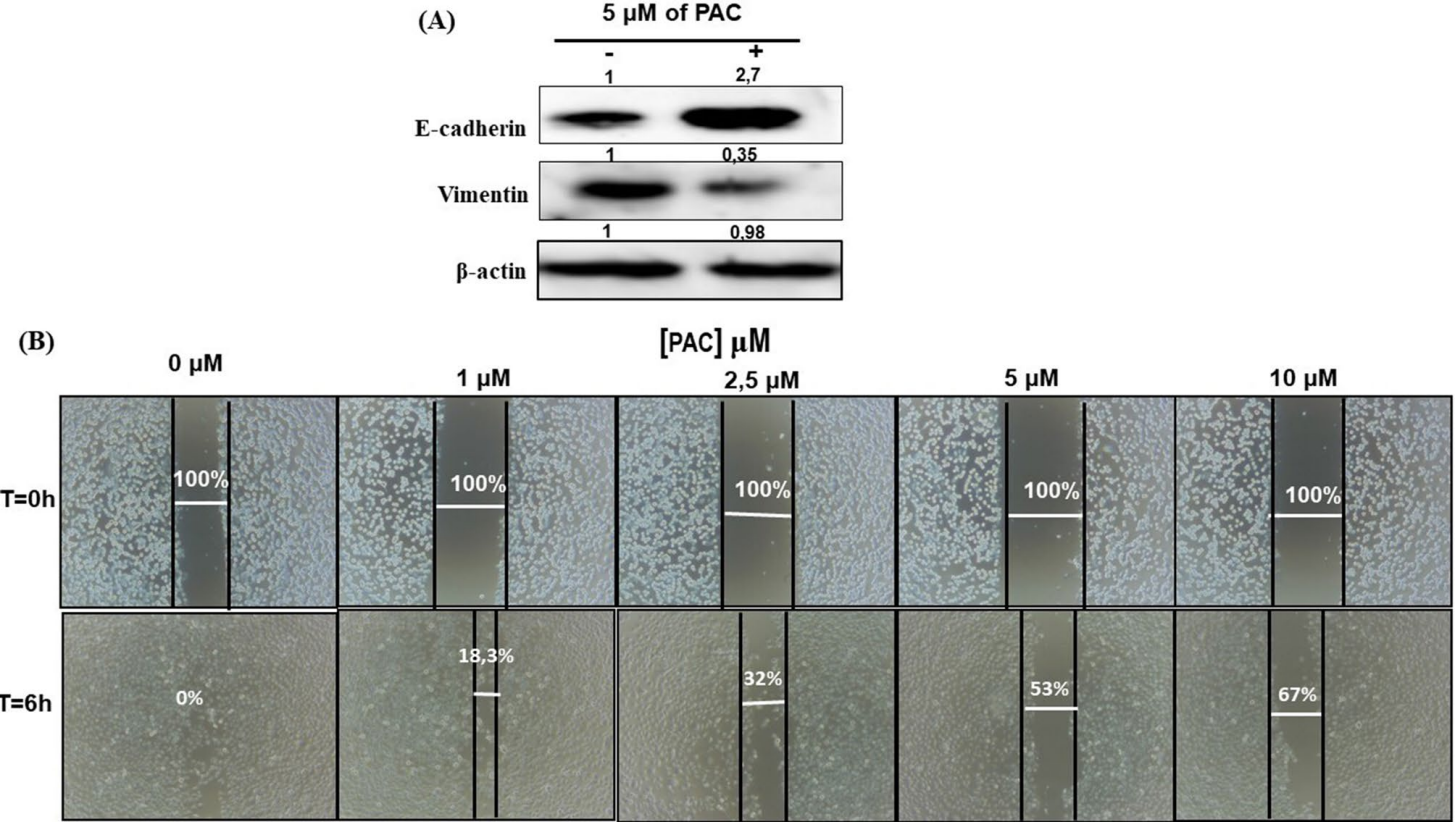
PAC suppresses the process of epithelial-to-mesenchymal transition and inhibits cell migration in oral cancer cells. (**A**) PAC at 5 µM inhibits the EMT in oral cancer cells by increasing E-cadherin expression, while vimentin expression was decreasing (n = 3). (**B**) Wound Healing (Scratch) Assay: after 6 h of treatment, PAC dose-dependent inhibits ca9-22 cell migration (n = 3). All Blots derive from the same experiment and were exposed at same time of phosphor imager exposition.

In Fig. 6A, we demonstrated that PAC at 5 µM inhibits the EMT in oral cancer cells, as shown by the increase of E-cadherin, while vimentin expression was decreasing. Metastatic cancer is also closely associated with cell migration. In this sense, we investigated the effect of various PAC concentrations on Ca9-22 cells migrating under treatment by using the Wound Healing (Scratch) Assay. As shown Fig. 6B, PAC at various concentrations was able to decrease the migration of oral cancer cells. Indeed, after 6 h of treatments with PAC, 100% of the scratch area was covered due to cell migration, in the controls, while less than 47% and 33% of the scratched area being covered following exposure to 5 µM or 10 µM, respectively.

### ROS production during treatments with PAC in oral cancer cells

**Figure 7:**
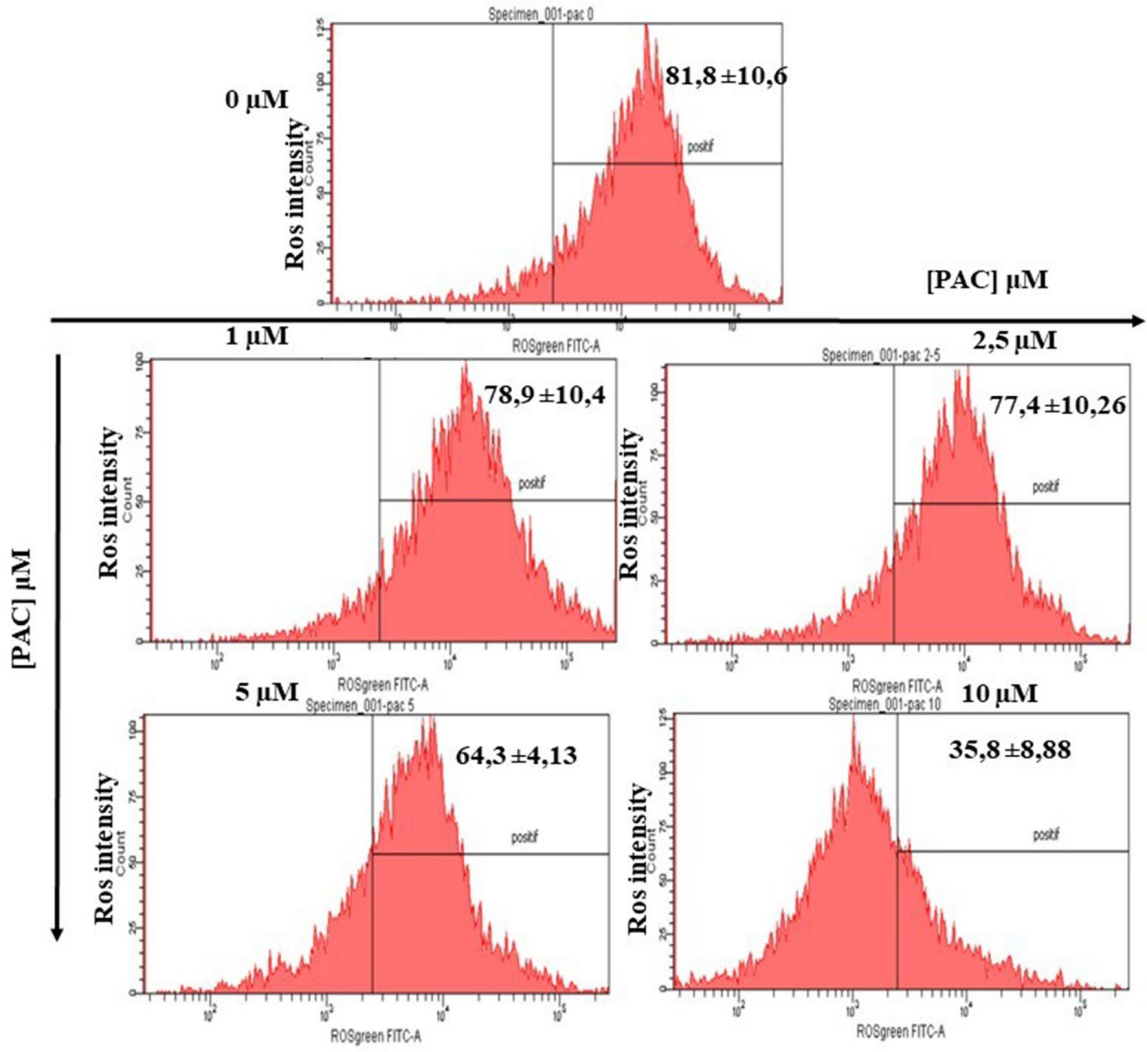
ROS production during treatments with PAC in oral cancer cells. Flow cytometry assay to evaluate the intracellular ROS accumulation after PAC-treatment for 24 h with PAC (n = 3).

Our data shows that the intracellular ROS accumulation induced by the treatment for 24 h with PAC was evaluated using a flow cytometer (Fig. 7). The percentage of ROS ( +) is low with increased concentration of PAC as treatment. In addition, the percentage of positive ROS staining in untreated cells was 81.8% ± 10.6% decreases to 64.3% ± 4.13%, *p* = 0.02 and to 35.8% ± 8.8%, *p* = 0.002 respectively when the cells were treated by 5 and 10 µM of PAC. However, any difference was observec when the cells were treated at low concentration of PAC (1 and 2.5 µM). We, therefore, suspect that PAC acts as a powerful antioxidant by producing intracellular glutathione (GSH).

### GSH generation during treatments with PAC in oral cancer cells

**Figure 8:**
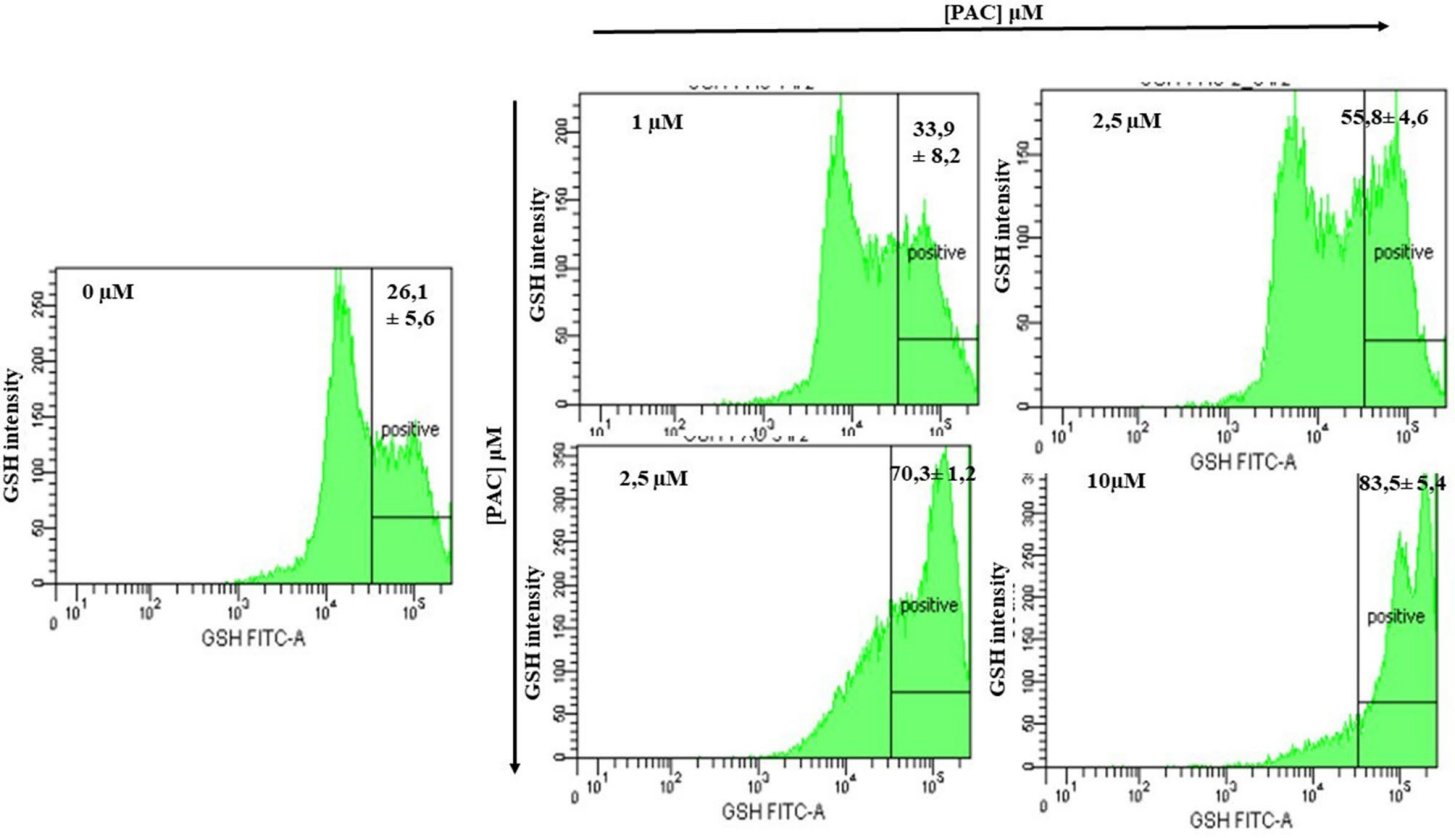
PAC acts as a powerful antioxidant by producing intracellular glutathione (GSH). To evaluate the levels of intracellular GSH, fluorescence intensity was determined by Flow Cytometry assay and the percentage of GSH-positive cells was calculated (n = 3).

Figure 8 shows that PAC strongly increase the GSH expression with 2.5 µM (55.8% ± 4.6%, *p* = 0.01compared to controls (26.1% ± 5.6%). The percentage of GSH positive is going up, from 26.1% with non-treated cells to 70.3% ± 1.2%, *p* = 0.004 and 83.8% ± 5.4%, *p* = 0.004), respectively after cell exposure to 5 µM or 10 µM of PAC, for 24 h. PAC is then confirmed to play a crucial role in producing GSH, an antioxidant.

### Mitochondrial membrane potential is reduced during treatments with PAC in oral cancer cells

**Figure 9:**
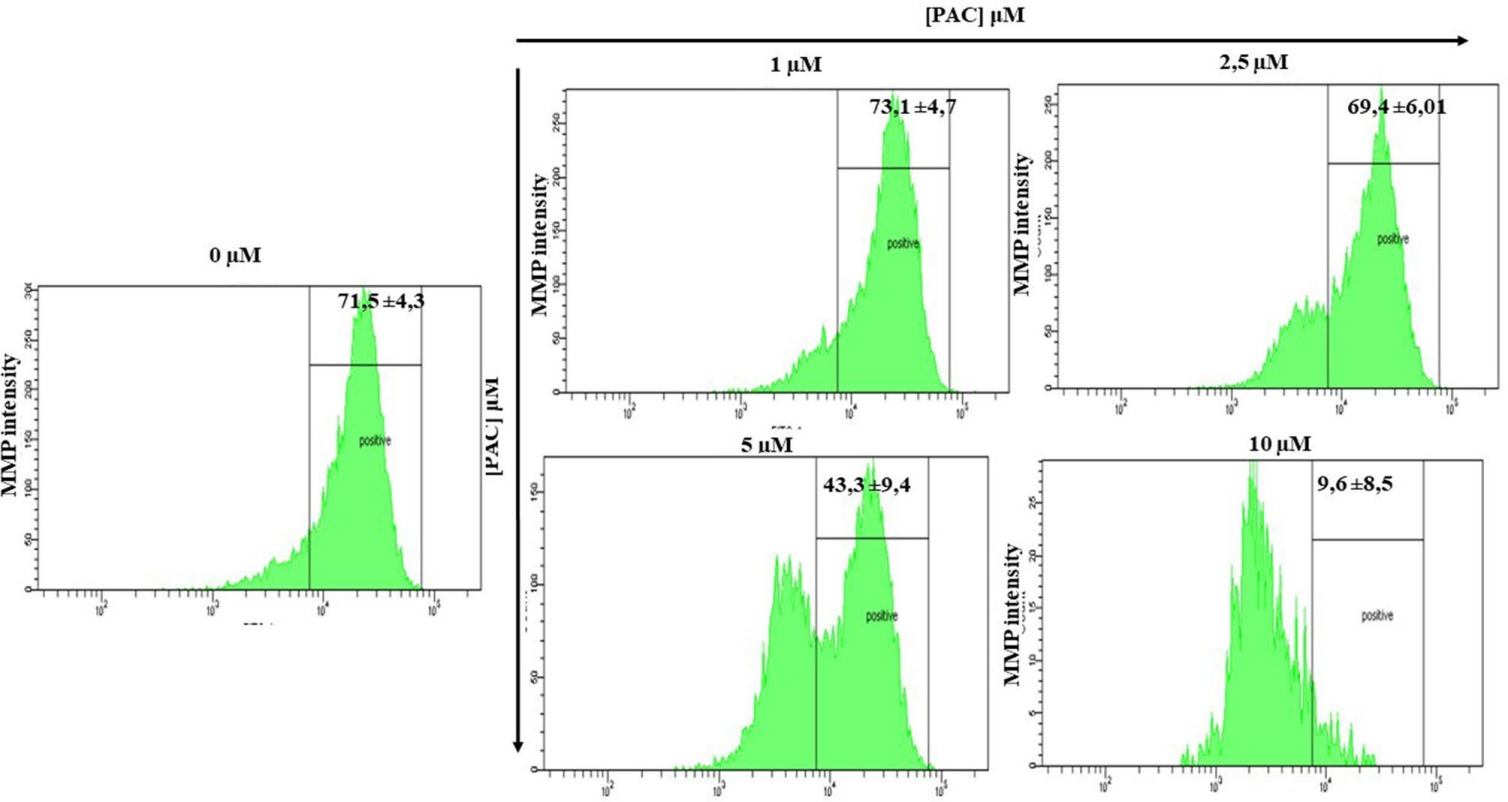
PAC- treatment reduces mitochondrial membrane potential (MMP) in oral cancer cells. The MMP expression was measured by the MitoProbe DiOC_2_(3) Assay Kit for Flow Cytometry (n = 4).

The MMP expression that is reduced by PAC was measured by the MitoProbe DiOC_2_(3) Assay Kit for Flow Cytometry. As shown in Fig. 9**,** there is a concentration-dependent effect of PAC on MMP intensity in Ca9-22 cells. Indeed, the MMP intensity decreases from 71.5% ± 4.3% for controls to 4.3% ± 9.4%, *p* = 0.01 and 9.6% ± 8.5%, *p* = 0.003 for Ca9-22 treated cells with 5 µM or 10 µM respectively for 24 h. In addition, any significant difference was observed when the cells were treated by PAC at 1 and 2.5 µM (Fig. 9).

### Mitochondrial superoxide production during treatments with PAC in oral cancer cells

**Figure 10:**
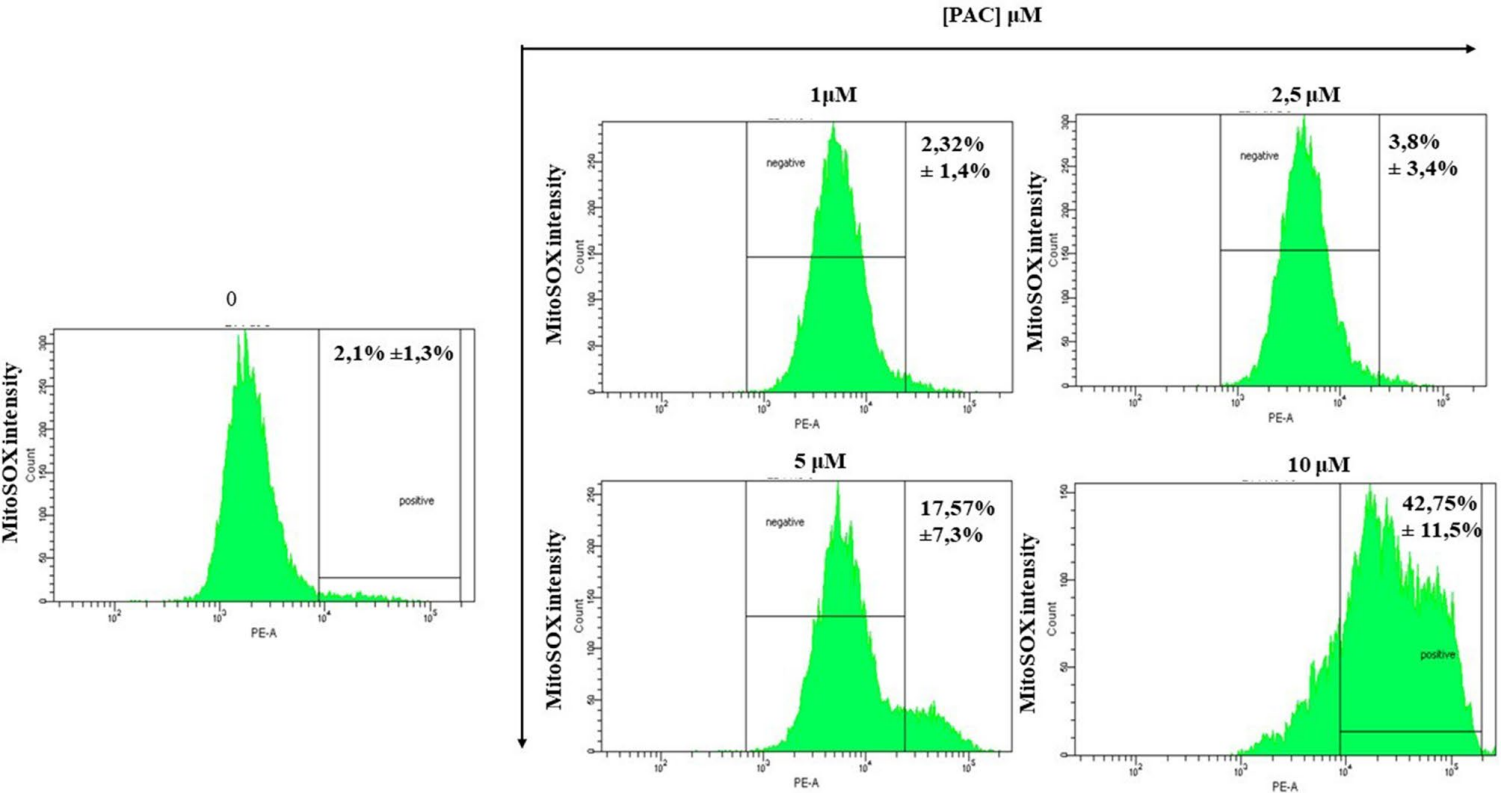
Mitochondrial superoxide production during treatments with PAC in oral cancer cells by flux cytometry. PAC-treated cells (1µ M, 2.5 µM, 5 µM or 10 µM) were incubated for 30 min with 5 μM MitoSOX at 37 °C and in 5% CO_2_ and were analysed by means of flow cytometry with “LSRII” or “CantoII” (BD Biosciences) (n = 3).

Figure 10 is corresponding to the concentration effect of flow cytometry patterns when oral cancer cells are PAC-treated. Our results show that the percentage of MitoSOX ( +) among Ca9-22 cells under treatment with PAC at 5 µM or 10 µM for 24 h is higher than controls. The percentage of MitoSox ( +) cells increases from 2.1% ± 1.3% in untreated cells to 17.57% ± 7.3%, *p* = 0.01 and to 42.75% ± 11.5%, *p* = 0.003 respectively when the cells were treated by 5 and 10 µM of PAC concentration (Fig. 10). The key role of PAC inducing mitochondrial superoxide production was clarified.

## Discussion

Conventional oral cancer treatments rely on surgery, along with radiation therapy (external beam radiotherapy and/or brachytherapy), as well as coadjutant therapy (chemotherapy with agents like cisplatin), it still involves high economic costs and highly damaging options^30,31^. To reduce this burden, plant products as complementary and alternative therapies could become solutions. Some recent discoveries demonstrated the superiority of the plant world to prevent cancer^32,33^. Several research studies over the last century have revealed several important functions of curcuminoids. And it has been shown that curcumin has a capacity to influence multiple natural targets and seemed to have activity against different diseases^12,15^. Although it was evaluated with success for widespread biological activities, those of concern are its low bioavailability and rapid metabolization encouraged researchers to look for new synthetic analogs^23,34^. In a search for novel compounds active against cancer, PAC was found out. This curcumin analog demonstrated that it has a great potential as an anti-breast cancer agent^21,24^. Until today, there had been no available study investigating the properties of PAC to be a possible complementary treatment for oral cancer. Our goal was to examine its inhibitory effects, on cells that proliferate or are in apoptosis, as well as the mechanisms of action involved as an interesting avenue to develop treatments for oral cancer and study its potential to be an active supplement ingredient in some drugs and products for mouth cleaning. Our data shows that PAC is exhibiting preferentially ability to kill oral cancer cells. Our data are supportive to those reported by Al-Howail et al. (2016) and Al-Hujaily et al. (2011) showing that PAC has similar effect against breast cancer^21,24^. PAC decrease the cell proliferation through the deregulation of the cell cycle. Indeed, PAC treated cells showed high expression of CDKIs including p21, p27, p16 or p53 and Rb. It is well known that these genes suppress tumors, known for being inactive in most types of cancer and also for inhibiting genes with oncogenic functions like cyclin D1 or c-Myc. These molecules have a history of playing a major role in the cell-cycle arrest at G2/M, and ultimately induce inhibition of oral cancer cell proliferation. This is mediated by inhibiting several signaling pathways involved in cancer such as MAPK, Wnt and NF-κB. The same results were reported by Al-Hujaily et al. (2011) and Al-Howail et al. (2016) reporting that PAC could be considered as a powerful nontoxic new chemotherapeutic agent against ER-negative tumors by triggering breast cancer cell apoptosis more than curcumin^21,24^, downregulating ERα, c-Myc, cyclin D1^24^ and inducing cell-cycle arrest at G2/M of this cancer cells^21^. Also, our results demonstrate that PAC strongly induces oral cancer cell apoptosis mediated via the mitochondrial pathway by increasing the Bax/Bcl-2 protein expression ratios, activating caspase-3/9 and cleaving PARP-1. We conclude that the PAC anti-oral cancer properties were controlled by impairing pro-survival signaling pathways and activating apoptosis pathways such as ERK1/2 activation, but not MEK1/2, AKT activation, NF-κB^35,36,37^ and β-catenin^38^, known to be implicated in tumor progression and as well for their resistance to chemo- and radiotherapy in several cancer types. Indeed, it is clearly reported that several oncoproteins such as cyclin D1 and p21 are strongly destabilized by inhibition of ERK1/2 activation. Wnt/β-catenin is the key oncogenic signaling pathway found to be highly active in various types of cancer, including oral cancer^38,39^. Similar observation was reported on breast cancer cells being treated with PAC^21,24^. Therefore, the strong PAC-dependent apoptosis could be of great value for inhibiting of oral cancer cell proliferation and inducing their death. In a recent study carried out by Al-Qasem et al. (2016), they have published the same effect of PAC on colon cancer cells. They concluded that PAC exhibits anti-colon cancer properties that have potential, by targeting cyclin D1 and suppressing JAK2/STAT3, AKT/mTOR and MEK/ERK signaling pathways as well as their common downstream effector which is cyclin D1^25^. We suggest that PAC suppresses the activation of MAPK and the induction of caspase cleavage, leading to the downregulation of several MAPK targets including cyclin D1, p53, and caspases, as Bax, Bcl-2 and cytochrome C. All these molecules are playing key roles in oral tumorigenesis^40–42^. In other hand, we demonstrated that PAC promoted oral cancer cell autophagy and reduced oxidative stress via ROS production. Apoptosis and autophagy are two important cellular processes play major roles in determining cellular fate^43^ particulary in cancer development.. Cancer is defined as the balance between cell proliferation and apoptosis. Developing a new drug that activates apoptosis in cells could be of great value to target the screening of therapeutic agents against cancer, specifically when we are focusing to find out compounds able to affect mitochondria, which play an important role in controlling this cellular suicide. The reduction of the ROS intracellular by PAC treatment can be explained by the fact that, cancer cells generate high levels of reactive oxygen species (ROS), as an unavoidable consequence of metabolism. These are considered as potentially harmful and usually linked to cancer progression. In our study, we demonstrated that PAC decrease intracellular ROS production and activate GSH, an antioxidant. We suggested that hyper proliferation of Ca9-22 cells is accompanied by a high rate of ROS production, but when treated with 10 μM of PAC, they adapted under similar normal conditions; they achieve this by increasing their antioxidant status in order to optimize ROS-driven proliferation, as reported by Dodson et al. (2019)^44^. Most publications indicate that an increasing GSH synthesis leads to improving NADPH production and trans-repression of pro-oxidant genes like NOS2 and COX2^45^. These results are in disagreement with several current research studies focusing on the use of anticancer treatments having an effect while apoptosis in cancer cells is induced by increased production of reactive oxygen species (ROS). However, our findings are also in concordance with others recent studies reporting that increase in cellular O2– and H2O2 by NADPH oxidase^46^ are leading the stimulation of cancer cell proliferation by ROS-sensitive AKT/ERK signaling pathways and the reduction of cancer cell growth by blocking this enzyme^47^; Dikalova et al. (2010) by using mitoTEMPO, they significantly reduced ROS in cells and inhibited NADPH oxidase activity^46^. As for Nazarewicz et al. (2013), they also reported that mitochondrial ROS scavenging by mitoTEMPO can block ROS-sensitive signaling pathways in cancer cells, cancer metabolism and induce cell death^48^. Our data suggests that diminishing ROS production, by PAC-treated oral cancer cells, is altering cell signaling mediated by ROS-sensitive AKT, ERK1/2, and p38 as well as inducing cell death. The PAC-attenuation effect from these signaling pathways was the principal factor leading to dramatic changes in oral cancer cell metabolism. As well, its anticancer activity toward mitochondria-targeted antioxidants (GSH) is closely associated with inhibition of ROS-sensitive AKT/ERK survival signaling pathways and glycolysis in human oral cancer cells. GSH is our body’s antioxidant powerhouse that is protecting our cells from free radical damage. It has been well reported that antioxidants have beneficial health effects at physiological concentrations^49^. However, in normal situations, the basal level of ROS is crucially good to cell viability. It may prevent oxidative stress by maintaining the balance in ROS homeostasis, especially in cancer cells known for having much faster metabolism and higher ROS levels compared to our controls^49^. Inhibition of mitochondrial superoxide may represent a novel, specific anticancer treatment strategy reducing side effects that are cytotoxic. Indeed, our work as well shows that PAC reduced mitochondrial ROS and decreased MMP. Oxidative stress is also known to induce disruption of the latter^50^ MMP is occurring to play a key role in mitochondrial homeostasis through selective elimination of those that are dysfunctional^51^. But MMP was considered as the central bioenergetic parameter for transporting ions and proteins that are indispensable for the control of normal mitochondrial function and are correlating with cell differentiation, tumorigenesis and being malignant^52^. Many other studies reported that carcinoma-derived cells possess a higher mitochondrial membrane potential than normal ones^53,54^ In addition, Schieke et al. (2008)^55^ published that higher MMP is linked with a tumorigenic property^55^. After inhibition of MMP by rapamycin treatment on stem cells having a high protonmotive force, its tumorigenicity decreased drastically. Moreover, our findings indicate that PAC induces intracellular ROS, generates mitochondrial superoxide and diminishes MMP leading to the release of cytochrome C in mitochondria and to the enhancement of oxidative stress in oral cancer cells.

## Conclusion

**Figure 11:**
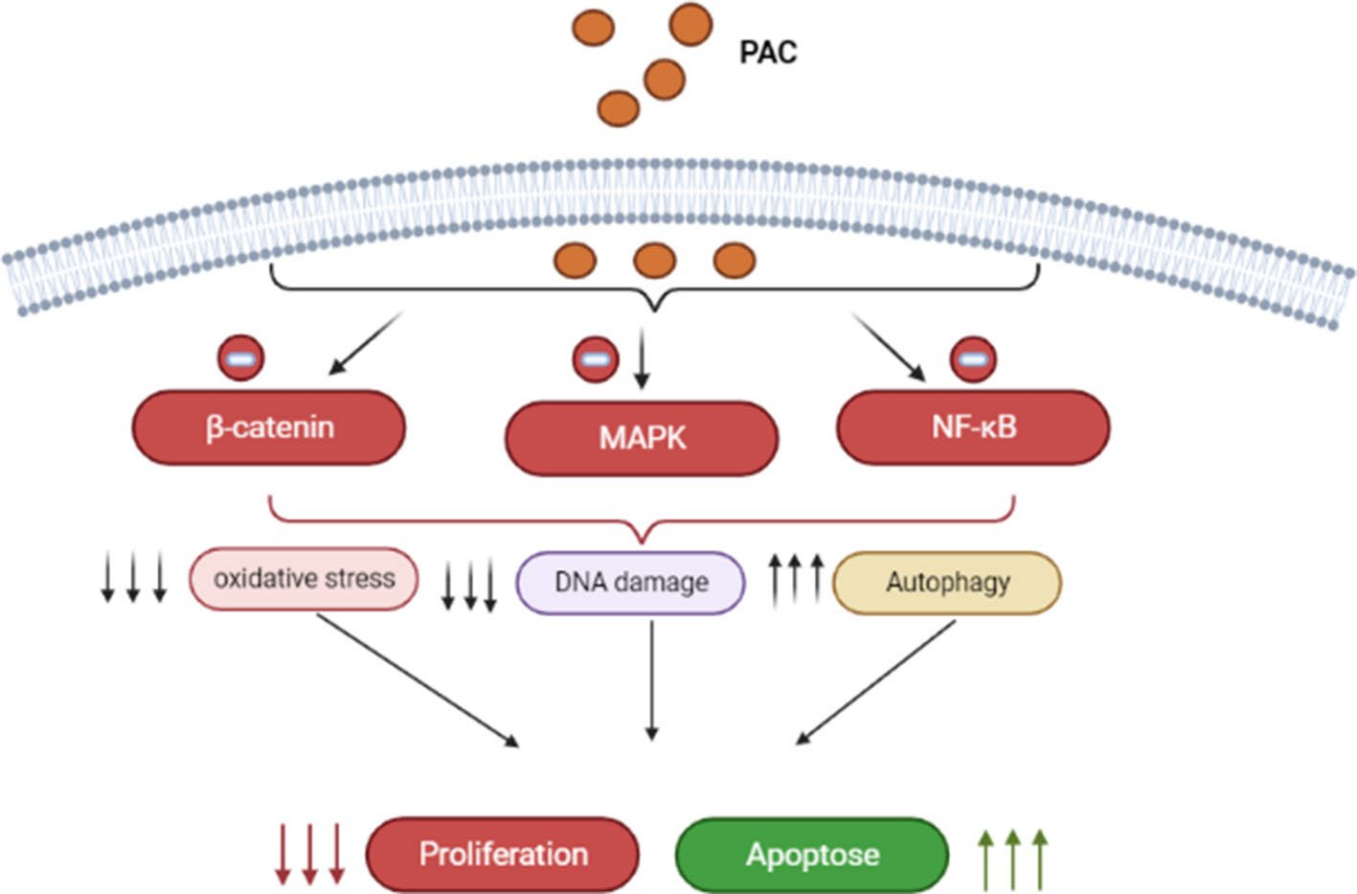
A final summary schematic representation of PAC mechanism of action.

The present study examined the antioral cancer and preferential killing effects of PAC, new analog of curcumine. Our results demonstrate the PAC preferential killing against oral cancer cells without effects on normal oral cells involving the regulation of proliferation, apoptosis, autophagy and oxidative stress. Therefore, PAC inhibits intracellular ROS, increased MitoSOX, diminished MMP, and PAC plays a crucial role in producing GSH, an antioxidant. With its preferential killing ability, PAC may provide a potential for improving oral cancer therapy without a cytotoxic side effect to normal oral cells (Fig. 11). The future studies will be to combine cisplatin with this new curcumin analog (PAC), to normalize cisplatin side effects and potential drug resistance on oral cancer.

## Supplementary information

Supplementary Information.

## Abbreviations

Bcl-2: B-cell lymphoma 2
CDKI: Cyclin dependent kinase inhibitor
DMSO: Dimethyl sulfoxide
EMT: Epithelial-to-mesenchymal transition
FBS: Fetal bovine serum
FCS: Fetal calf serum
GSH: Glutathione
GEC: Primary human gingival epithelial cells
LDH: Lactate dehydrogenase
MTT: 3-(4,5-Dimethylthiazol-2-Y1)-2,5-diphenyltetrazolium bromide
MEK/ERK: Mitogen-activated protein kinase/extracellular signal-regulated kinase
MMP: Mitochondrial membrane potential
NF-κB: Nuclear factor-kappa B
OC-SCC: Oral cavity squamous cell carcinoma
PAC: (3,5-*Bis* (4-Hydroxy-3-methoxybenzylidene)-*N*-methyl-4-piperidone)
PARP1: Poly(ADP-ribose) polymerase 1
ROS: Reactive oxygen species
SD: Standard deviation
