# Supplementary figures and images for: The curcumin analog (PAC) suppressed cell survival and induced apoptosis and autophagy in oral cancer cells
Source: Sci Rep. 2021 Jun 3;11:11701. doi: 10.1038/s41598-021-90754-x (PMC8175612; doi:10.1038/s41598-021-90754-x)

## Slide 1
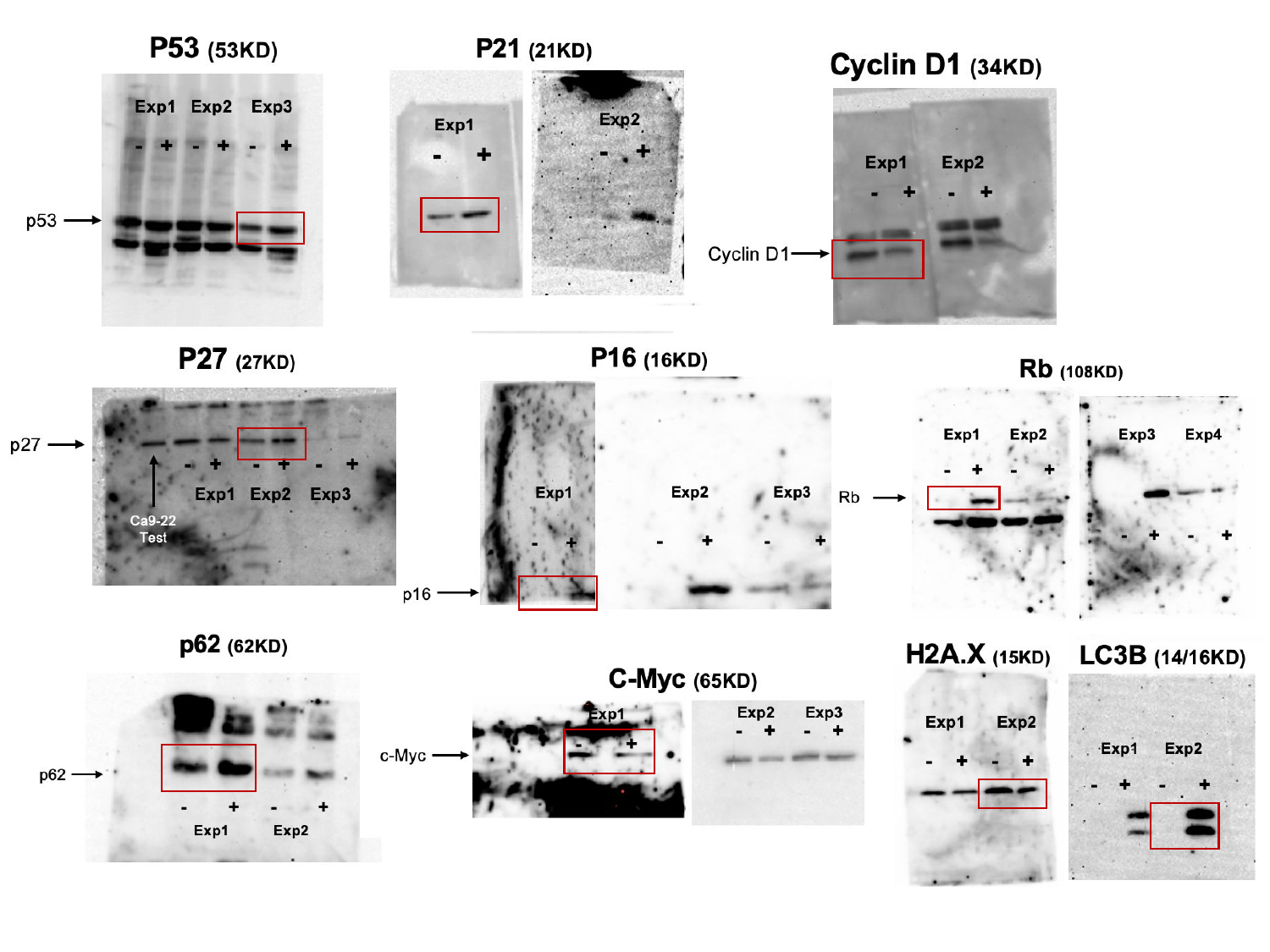

## Slide 2
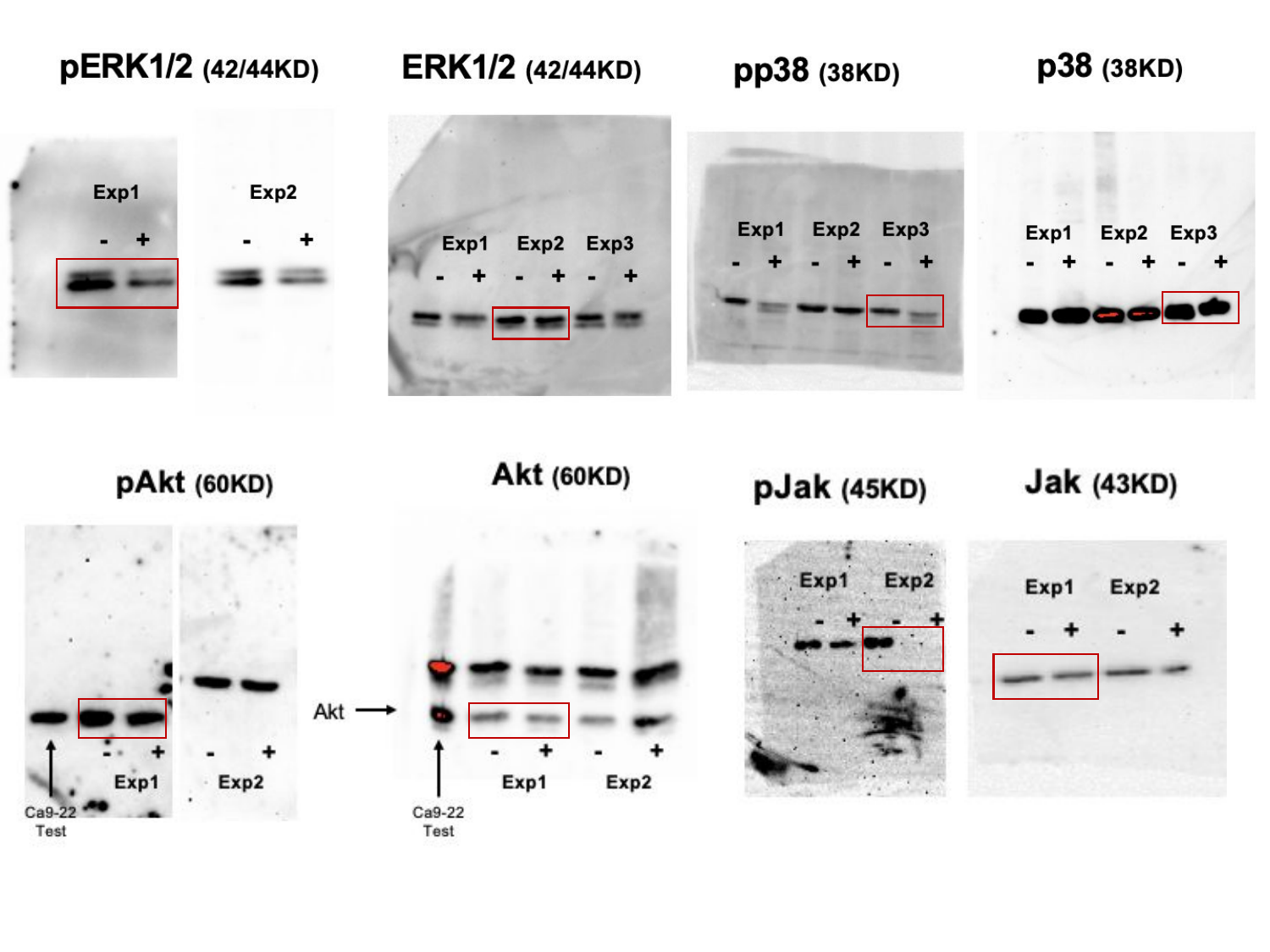

## Slide 3
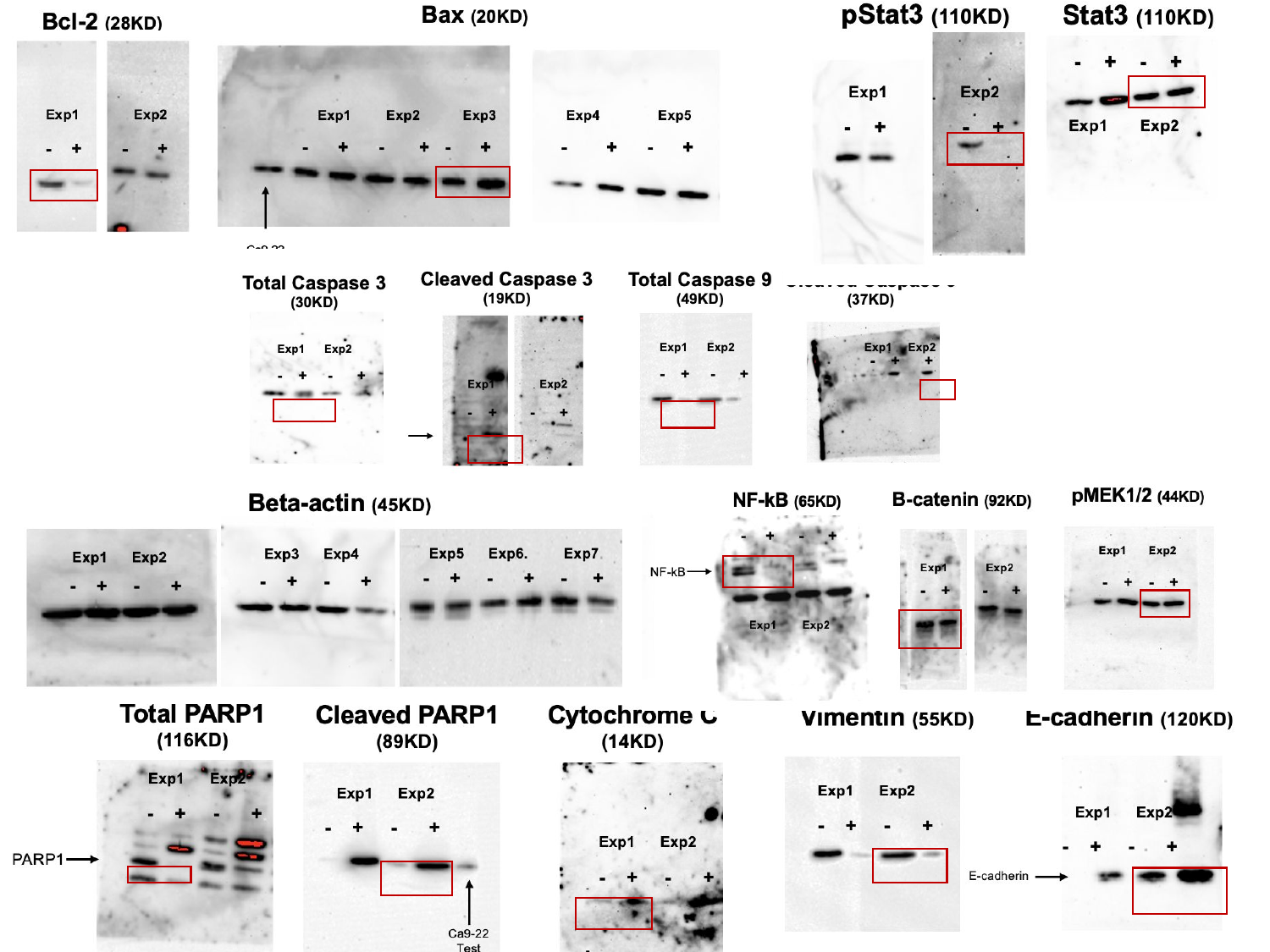

Supplement: Supplementary file 1 — Supplementary Information. [file 41598_2021_90754_MOESM1_ESM.pptx]
